# Sodium L-Aspartate Supplementation Improves Repeated-Sprint Performance

**DOI:** 10.3390/nu15245117

**Published:** 2023-12-15

**Authors:** Keiichi Yamaguchi, Nanako Hayashi, Daichi Sumi, Miho Ono, Tomonori Koizumi, Wataru Sato, Fumika Takeuchi, Yusuke Adachi, Kazushige Goto

**Affiliations:** 1 Graduate School of Sport and Health Science, Ritsumeikan University, Kusatsu 525-8577, Japan; 2 Japan Society for Promotion of Science, Tokyo 102-0083, Japan; 3 Research Center for Urban Health and Sports, Osaka Metropolitan University, Osaka 558-8585, Japan; 4 Ajinomoto Co., Inc., Kawasaki 210-8681, Japan

**Keywords:** aspartate, amino acid, sprint performance, acid-base balance

## Abstract

Aspartate supplementation has been reported to improve endurance performance by facilitating the tricarboxylic acid cycle flux. The present study was performed to investigate the effects of aspartate supplementation on repeated-sprint performance and blood pH. Following an overnight fast, fourteen healthy males completed three sets of 10 × 6 s maximal sprints after consuming sodium L-aspartate (ASP) or placebo (PLA), in a double-blind manner. Both supplements were taken twice on each test day (2 × 4.5 g). Exercise performance (e.g., cadence and power output) and blood variables (e.g., pH and plasma amino acid levels) were measured. The ASP trial evidenced significantly higher plasma aspartate concentration during the first (ASP, 45.3 ± 9.2 μM; PLA, 6.1 ± 0.8 μM) and the second exercise sets (ASP, 24.2 ± 4.5 μM; PLA, 6.6 ± 0.9 μM) and peak cadence during the second set (ASP, 153 ± 3 rpm; PLA, 152 ± 3 rpm) compared with the PLA trial (all *p* < 0.05). The peak power output during the second exercise set (ASP, 743 ± 32 W; PLA, 734 ± 31 W; *p* = 0.060) and the blood pH immediately before (ASP, 7.280 ± 0.020; PLA, 7.248 ± 0.016; *p* = 0.087) and after the third exercise set (ASP, 7.274 ± 0.019; PLA, 7.242 ± 0.018; *p* = 0.093) tended to be higher in the ASP than in the PLA trial. In conclusion, ASP supplementation partially improved repeated-sprint performance (peak cadence during the second exercise set). However, it did not affect the mean power output.

## 1. Introduction

Aspartate is a non-essential amino acid that is a precursor of oxaloacetate, an intermediate of the tricarboxylic acid (TCA) cycle [1]. Aspartate supplementation improves the biochemical capacity for fatty acid oxidation by facilitating the TCA cycle flux [2], thereby enhancing oxidative phosphorylation and lowering the blood lactate concentration during submaximal exercise [3]. Several studies reported that aspartate supplementation increased the time to exhaustion during submaximal cycling by 15–50% [4,5,6]. For example, Wesson et al. [6] demonstrated that the cycling time to exhaustion at 75% of the maximal oxygen consumption ($\overset{\cdot }{V}$O_2max_) was significantly increased by potassium–magnesium–aspartate supplementation (87.6 ± 4.3 min) compared with the placebo condition (75.7 ± 11.9 min). Although several studies failed to find a beneficial effect of aspartate supplementation on exercise performance [7] and physiological responses [8], Trudeau [1] concluded in a review that aspartate supplementation improves endurance in humans. However, the effect of aspartate supplementation on high-intensity exercise performance, such as repeated-sprint ability (RSA), has not been explored.

In various team and racket sports, athletes are required to repeatedly engage in brief (≤10-s) bouts of maximal sprints with incomplete (≤60-s) recovery throughout the game. The ability to recover and reproduce power output during successive sprints is termed RSA (repeated-sprint ability) [9,10]. Superior RSA was associated with longer sprinting and high-intensity running distances during soccer matches [11] and better running and combat performance during rugby matches [12], suggesting that improving RSA would enhance match performance in team sports. During repeated-sprint exercise, energy derived from anaerobic glycolysis and phosphocreatine (PCr) is predominantly consumed during the first sprint (approximately 40% from glycolysis and 46% from PCr), and the contribution of oxidative metabolism increases progressively as sprints are repeated; oxidative metabolism contributes approximately 40% of energy supply during the final repetition of 10 × 6 s maximal sprints) [10]. The lack of energy supply (e.g., PCr and oxygen) and metabolite (e.g., inorganic phosphate and hydrogen ion [H^+^]) accumulations are proposed to be key limiting factors of RSA [10]. Aspartate supplementation may improve RSA by enhancing the TCA cycle flux. The cycle acceleration would increase aerobic metabolism, mitigating metabolite accumulations and subsequent acidosis, leading to improving RSA. However, the impact of aspartate supplementation on RSA has not been elucidated so far.

In the present study, we determined the effects of aspartate supplementation on performance and blood pH during a repeated-sprint exercise. We hypothesized that aspartate would mitigate blood pH reduction and improve the RSA relative to the placebo (PLA).

## 2. Materials and Methods

### 2.1. Study Design and Participants

The present study was a randomized, double-blind, placebo-controlled, crossover study. A total of 15 healthy active males were recruited in the present study. Fourteen participants completed all experiments (means ± standard errors; age 22.3 ± 0.3 years, height 172.1 ± 1.7 cm, weight 65.4 ± 2.2 kg), while a participant was excluded because he could not complete all the experiments. We recruited individuals who (1) were aged between 20 and 29 years, (2) were healthy Japanese males, (3) had exercise habits, and (4) were capable of independent decision-making and the provision of written informed consent. The exclusion criteria were as follows: the participants who (1) were considered to have difficulty in exercising due to medical or surgical diseases, (2) were participating in other research or participated in a study requiring the chronic intake of medicines or specific foods within the preceding month, and (3) had undergone blood tests or given blood, with a collection of more than 200 mL of blood, within 12 weeks of this study. All participants were non-smokers and were asked to avoid intense exercise, caffeine, alcohol, and supplements for 24 h before each experiment. They were not accustomed to the repeated-sprint exercise with maximal effort on the cycle ergometer, so the familiarization was conducted before the main experimental trials. The diet pattern on the day before experimental trials was not controlled, but the participants were asked to maintain regular dietary habits throughout the study. Participant recruitment into the study began in June 2019 and ended in July 2019. The last participants completed a cross-over trial in August 2019. This study was conducted in accordance with the Declaration of Helsinki. All participants were given an overview, potential risks, and benefits of the present study and provided written informed consent. The study was approved by the Research Ethics Committees of Ritsumeikan University, Japan (BKC-IRB-2018-071), and Ajinomoto Co. Inc. (2018-013). It was registered in the University Hospital Medical Information Network Clinical Trials Registry (UMIN000037306).

### 2.2. Experimental Overview

All participants visited the laboratory three times. On the first visit, they were familiarized with the experimental procedure and the repeated-sprint exercise. First of all, the physical characteristics (body height and weight) were measured, and the seat height was adjusted for each participant to ensure a slight flex in the knee at the lowest pedal position. The seat height was recorded and replicated in all experimental trials. After 5 min warm-up exercise (3 min low-intensity cycling [1.0 kp, 90 rpm] followed by three 6 s sprints at 60%, 80%, and 100% of the perceived maximal effort), they performed two sets of 10 × 6 s maximal cycling sprints (at a pedal load of 7.5% of body weight) interspersed with 40 s active recovery on an electromagnetically braked cycle ergometer (Power Max VⅢ; Konami Corp., Tokyo, Japan).

On the second and third visits, the participants engaged in two trials with aspartate or PLA supplementation scheduled in a double-blinded crossover manner. The trial order was randomized and counterbalanced. The two trials were separated by 1 week. One serving (4.5 g) of the aspartate supplement consisted of 4.0 g sodium L-aspartate (ASP) and 0.5 g food additives such as flavoring, and the PLA supplement consisted of 3.7 g maltitol and 0.8 g food additives. The test product was packed in single aluminum sachets. Both supplements were granulated and taken with 200 mL water twice on each trial day (25 min before and immediately after the first exercise set). Since a pilot study revealed that the blood aspartate concentration reached the peak value 25 min after consuming the supplement, we decided to give the supplements approximately 25 min before the first exercise set and again immediately after the first exercise set to maintain a high level of blood aspartate throughout the entire exercise.

**Figure 1:**
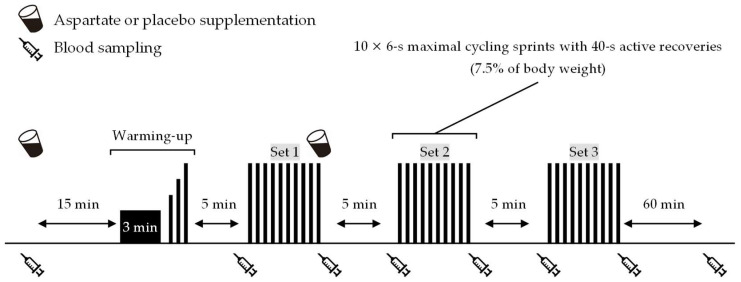
Experimental overview.

Figure 1 shows an overview of the experiment. Participants came to the laboratory at 8:00 or 9:30 a.m. (at the same time within participants) on trial days after an overnight fast. After a 10 min rest on chairs, a cannula was inserted into the antecubital vein, and baseline blood samples were taken. Then, they consumed one serving of the ASP or PLA supplement. They rested again for 15 min on chairs, followed by a 5 min warm-up consisting of 3 min low-intensity cycling (1.0 kp, 90 rpm) and three 6 s sprints at 60%, 80%, and 100% of the perceived maximal effort. A wireless heart rate (HR) monitor and a mask were attached to monitor cardiorespiratory data during a 5 min rest. Participants then performed the repeated-sprint exercise (three sets of 10 × 6 s maximal cycling sprints with 40 s active recovery between sprints and 10 min passive rest between sets) on an ergometer (Power Max VⅢ; Konami Corp., Tokyo, Japan). The pedal load was 7.5% of the body weight during sprints and zero during active recovery. Blood samples were collected immediately before and after each exercise set. After performing the final sprint of the first exercise set, the participants again consumed one serving of the ASP or PLA supplements. Following completion of the repeated-sprint exercise, participants rested for 60 min on chairs, and blood samples were collected again.

### 2.3. Measurements

#### 2.3.1. Repeated-Sprint Performance

During each sprint, the peak cadence and peak and mean power outputs were recorded. The fatigue index (FI) was calculated as a percentage reduction of mean power output from the best (MP_best_) to the worst (MP_worst_) sprints using the following equation, where MP_best_ and MP_worst_ are in W.

FI (%) = 100 × (MP_best_ − MP_worst_)/MP_best_

#### 2.3.2. Perceptual Response

The perceived exertions for breathing (RPE_breath_) and legs (RPE_leg_) were measured using a 10-point scale [13] immediately after each exercise set and averaged throughout the entire exercise.

#### 2.3.3. Cardiorespiratory Data

The HR was measured and recorded every second during the repeated-sprint exercise using a HR sensor (H2; Polar Electro Oy., Kempele, Finland) and a wireless monitor (RCX5; Polar Electro Oy., Kempele, Finland), and the mean HR was calculated for each exercise set. The HR sensor was attached with an elastic band on the chest, and the wireless monitor was used to observe the HR continuously throughout the exercise. Oxygen consumption ($\overset{\cdot }{V}$O_2_), carbon dioxide production ($\overset{\cdot }{V}$CO_2_), and minute ventilation ($\overset{\cdot }{V}$E) were monitored during all exercise sets using an automatic gas analyzer (AE-310S; Minato Medical Science Co. Ltd., Tokyo, Japan). The mask was attached on the face immediately before each exercise set and removed immediately after completion of each set. These variables were measured breath-by-breath and averaged for each exercise set. Before each experimental trial, prescribed calibration was conducted using gases containing known concentrations of O_2_ and CO_2_. A volume sensor of an automatic gas analyzer was also calibrated using a 2 L syringe.

#### 2.3.4. Blood Sampling and Analysis

Blood samples were collected eight times through each trial (at baseline, immediately before and after each exercise set, and 60 min after exercise completion) into 15 mL and 2.5 mL syringes via a cannula inserted into the antecubital vein. Immediately after blood sampling, 12 mL of blood was transferred into an ethylenediaminetetraacetic acid-containing tube (6 mL) or a serum separation tube (6 mL). Glucose and lactate concentrations in the remaining blood samples were measured immediately using a glucose analyzer (FreeStyle Freedom Lite; Nipro Corp., Osaka, Japan) and a lactate analyzer (Lactate Pro 2; Arklay Inc., Kyoto, Japan), respectively. Plasma and serum samples were obtained by centrifugation for 10 min at 4 °C (3000 rpm, 1712× *g*) and stored at −80 °C for subsequent analysis. Plasma amino acids (aspartate, serine, glutamate, alanine, citrulline, phenylalanine, 3-methylhistidine, arginine, valine, leucine, isoleucine, total branched-chain amino acids [BCAA], and total amino acids) and catecholamines (adrenaline, noradrenaline, and dopamine) were analyzed using high-performance liquid chromatography, and serum albumin and myoglobin were assayed in a clinical laboratory (SRL Inc., Tokyo, Japan).

The blood pH, bicarbonate (HCO_3_^−^) concentration, and base excess were determined immediately after the blood collection into 2.5 mL syringes using a blood gas analyzer (OPTI CCA-TS2; OPTI Medical Systems Inc., Atlanta, GA, USA). The blood gas analyzer was calibrated using prescribed solution before each experimental trial. We evaluated the base excess to confirm metabolic acidosis because both metabolic and respiratory acidosis contribute to a decrease in pH, but the base excess is affected by metabolic acidosis and not by respiratory factors. The hemoglobin (Hb) and hematocrit (Hct) levels were measured for the evaluation of plasma volume changes (ΔPV) using the following equation, where Hb is in g/dL and Hct is in % [14].

ΔPV (%) = 100 × ([Hbpre/Hbpost] × [100 − Hctpost]/[100 − Hctpre] − 1)

#### 2.3.5. Statistical Analysis

All data are presented as means ± standard errors, and 95% confidence intervals (CI) are also presented. The test–retest reliability for peak cadence and peak and mean power outputs during the first 6 s maximal sprint was calculated. The intraclass correlation coefficients were 0.96, 0.99, and 0.93, respectively; the coefficients of variation were 0.66%, 0.66%, and 2.68%, respectively. The principal analysis was conducted with the generalized linear mixed model (GLMM) using the “lme4” package (ver. 1.1.27.1) of R (ver. 4.1.2). The linear predictors, as fixed effects, were the trial type (ASP or PLA), timepoint, interaction (trial × timepoint), sequence (trial order), and period (first or second trial). The participants were the random effects. The following model was used:

Y = β_0(intercept)_ + β_1(trial)_ + β_2(timepoint)_ + β_3(trial×timepoint)_ + β_4(sequence)_ + β_5(period)_ + r_(random effect)_.

When evaluating the blood pH, HCO_3_^−^, and base excess, the GLMM was applied to two interval structures: (1) baseline and immediately before and after each exercise set, and (2) baseline and 60 min after exercise completion. Statistical significance was set to *p* < 0.05.

## 3. Results

### 3.1. Cadence, Peak, and Mean Power Outputs during Repeated-Sprint Exercise

**Figure 2:**
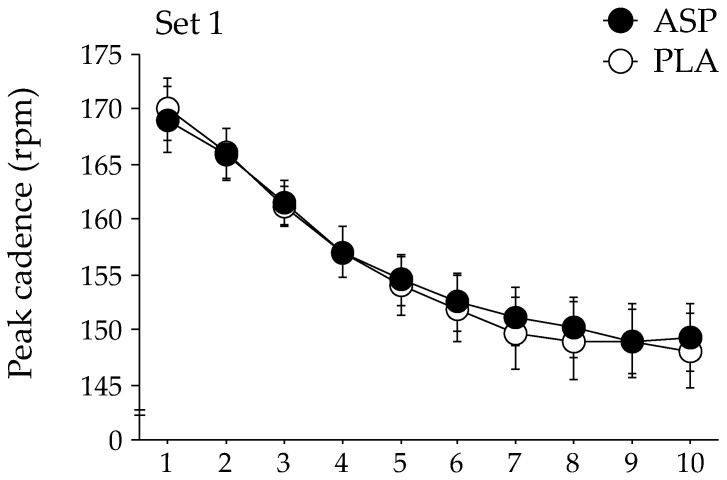

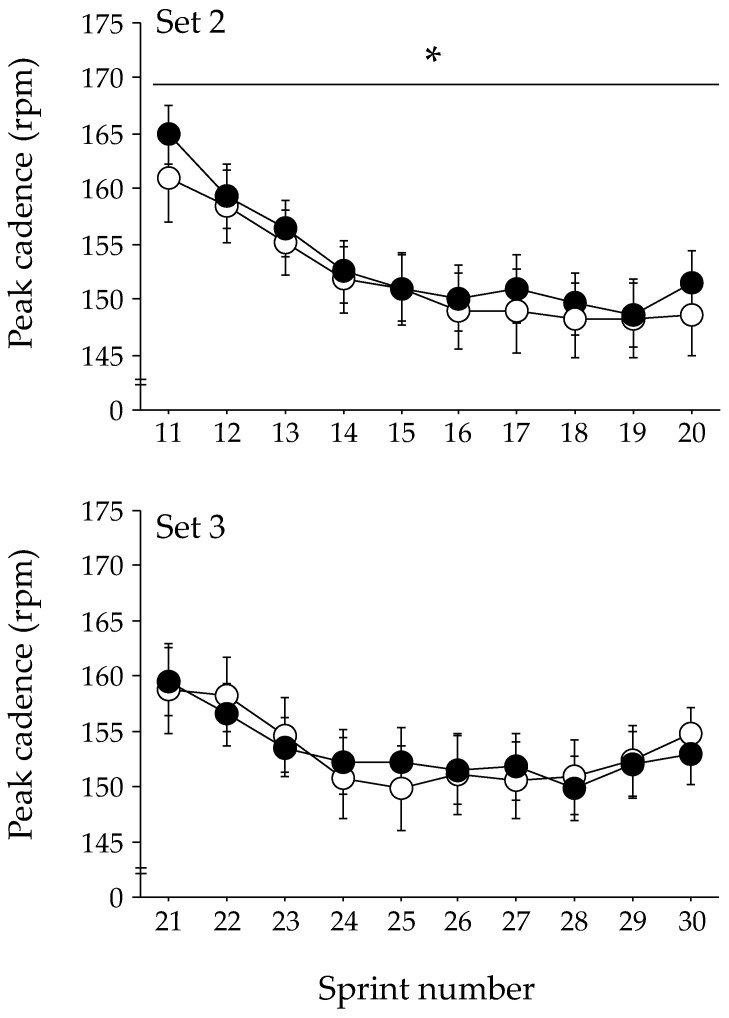
Changes in peak cadence during repeated-sprint exercise with aspartate (ASP) and placebo supplementations (PLA). Mean ± SE. * *p* < 0.05 between trials.

The changes in peak cadence during each exercise set of the repeated-sprint exercise are shown in Figure 2. The GLMM analysis indicated that the peak cadence was significantly higher in the ASP trial than in the PLA trial during the second exercise set (*p* = 0.048). No significant difference in the peak cadence between trials was observed during the first and third exercise sets. The peak power output during the second exercise set tended to be higher in the ASP trial than in the PLA trial (mean, 741 ± 32 W [CI: 679–804 W] and 734 ± 31 W [CI: 673–794 W], respectively); however, statistical significance was not attained (*p* = 0.060). The peak power output during the first and the third exercise sets were not different between the two trials. No difference between the ASP and PLA trials was observed at any timepoint in the mean power output (609 ± 28 W [CI: 554–664 W] and 611 ± 28 W [CI: 556–666 W], respectively).

### 3.2. FI, RPE, and Cardiorespiratory Data during Repeated-Sprint Exercise

There were no significant differences between trials in FI (ASP, 22.1% ± 1.7% [CI: 18.8–25.5%]; PLA, 20.9% ± 1.9% [CI: 17.1–24.7%]), RPE_breath_ (ASP, 4.4 ± 0.3 [CI: 3.9–5.0]; PLA, 4.8 ± 0.2 [CI: 4.4–5.2]), RPE_leg_ (ASP, 4.8 ± 0.3 [CI: 4.3–5.4]; PLA, 5.1 ± 0.2 [CI: 4.6–5.6]), HR, $\overset{\cdot }{V}$O_2_, $\overset{\cdot }{V}$CO_2_, and VE during any exercise set (Table 1).

### 3.3. Blood pH, Bicarbonate, and Base Excess Levels during Exercise

**Figure 3:**
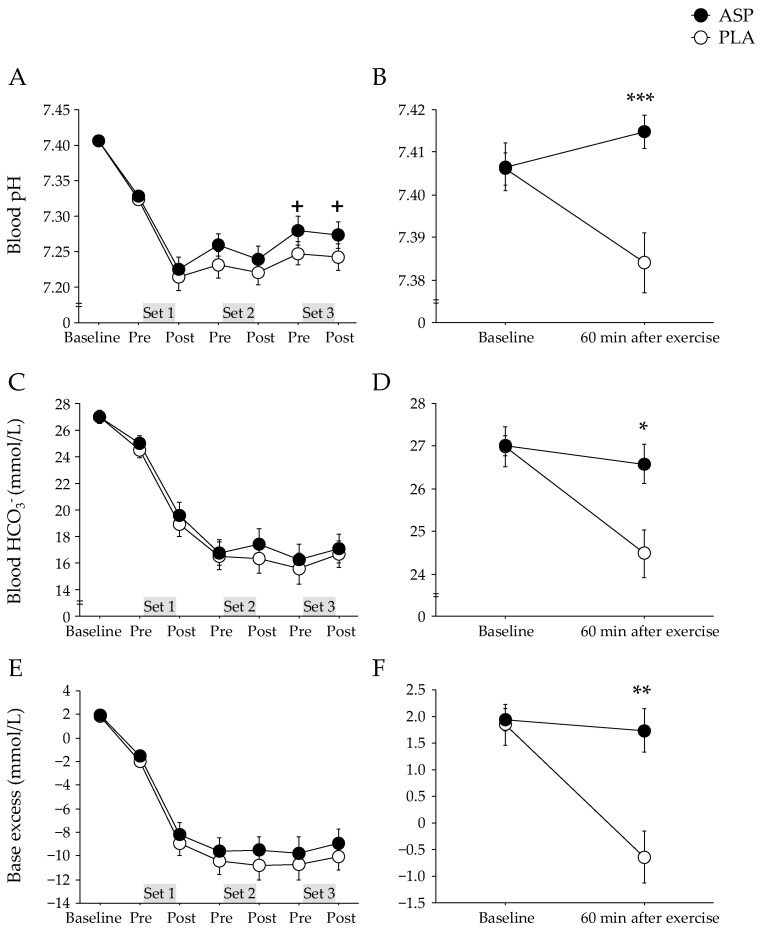
Blood pH (**A**,**B**), HCO_3_^−^ concentration (**C**,**D**), and base excess (**E**,**F**) immediately before and after each set and 60 min after completion of exercise with aspartate (ASP) and placebo supplementations (PLA). Mean ± SE. + *p* < 0.1, * *p* < 0.05, ** *p* < 0.01, *** *p* < 0.001 between trials.

The pH at baseline was 7.407 ± 0.006 [CI: 7.395–7.419] in the ASP and 7.406 ± 0.004 [CI: 7.398–7.414] in the PLA trials, and these levels were immediately reduced after repeated-sprint exercise. The ASP trial showed a trend for higher blood pH than the PLA trial (Figure 3A). The changes in blood HCO_3_^−^ and base excess did not differ between trials (Figure 3C,E). However, 60 min after exercise completion, the blood pH, HCO_3_^−^, and base excess were significantly higher in the ASP than in the PLA trial (*p* < 0.001, *p* = 0.013, and *p* = 0.001, respectively; Figure 3B,D,F).

### 3.4. Blood Parameters and Plasma Amino Acid Profile

No significant difference between the ASP and PLA trials was observed in the mean blood lactate (13.1 ± 1.1 and 13.3 ± 1.0 mmol/L) or glucose concentrations (103 ± 5 and 104 ± 5 mg/dL). Also, no significant difference was observed in the plasma adrenaline, noradrenaline, dopamine, and serum albumin concentrations (Table 2). Serum myoglobin concentration 60 min after exercise completion (ASP, 85.8 ± 10.3 ng/mL [CI: 65.5–106.1 ng/mL]; PLA, 82.6 ± 11.7 ng/mL [CI: 59.6–105.6 ng/mL]) and the mean ΔPV (ASP, −18.2% ± 1.7% [CI: −21.6~−14.9%]; PLA, −17.3% ± 0.9% [CI: −19.1~−15.6%]) were not different between trials.

Compared with the PLA trial, the ASP trial evidenced significantly higher plasma aspartate concentrations before the first (*p* < 0.001) and second (*p* = 0.012) exercise sets and significantly higher plasma total amino acid concentration before the second (*p* = 0.002) and third (*p* = 0.002) exercise sets (Table 3). Significantly higher plasma glutamine, alanine, citrulline, and phenylalanine concentrations were also observed in the ASP trial than in the PLA trial, while the plasma arginine and BCAA (valine, leucine, and isoleucine) concentrations did not differ between trials (Table 3).

## 4. Discussion

In the present study, we examined the effects of aspartate supplementation on performance and blood pH during repeated-sprint exercise in healthy males. Consequently, the ASP trial evidenced a smaller reduction in blood pH and increased peak cadence during the second exercise set than the PLA trial. To the best of our knowledge, this is the first study to indicate the effect of aspartate supplementation on RSA with concomitant data on acid-base balance.

Aspartate supplementation has previously been shown to decrease blood lactate concentration and increase fat oxidation during submaximal cycling at 100 and 150 W [3]. Moreover, aspartate supplementation extended the cycling time to exhaustion at individually chosen loads (HR: 170 bpm) [4]. In a review by Trudeau [1], aspartate supplementation is suggested to improve endurance performance in humans. However, no previous study has investigated the effect of aspartate supplementation on exercise performance during maximal intensity exercise, such as RSA. Our results suggested that aspartate supplementation partially improves the RSA. Attenuating blood pH reduction using supplements (e.g., sodium bicarbonate) is effective for improving moderate- to high-intensity exercise performance even in athletes [15,16,17], and therefore, the present findings would also be beneficial among athletes who are involved in team sports (e.g., soccer, basketball).

The high-intensity repeated exercise induces a fall in both blood and skeletal muscle pH [18]. The acidification would be due to the increases in hydrogen ion (H^+^) accumulation, which in turn, disturbs the state of equilibrium between acidity and alkalinity of body fluids. Metabolic acidosis is considered to be associated with the development of fatigue by impairing the release of calcium ions (Ca^2+^) from the sarcoplasmic reticulum, impeding glycolytic enzyme activity, and altering the strong ion difference, leading to reduced action potentials and muscle excitability. For this reason, buffering substances like sodium bicarbonate have been previously proposed as a performance-enhancing aid by reducing exercise-induced acidosis. In the skeletal muscle, aspartate is transformed into oxaloacetate by aspartate transaminase and then enters into the TCA cycle [1]. Thus, aspartate facilitates the cycle flux and improves the capacity for fatty acid oxidation [2], in turn reducing lactate and H^+^ accumulations [3]. In accordance with the hypothesis, the present study indicated that aspartate supplementation would mitigate the exercise-induced reductions of blood pH. Blood bicarbonate concentration and base excess also showed concomitant changes with blood pH during the repeated-sprint exercise, although they did not reach statistical significance. Thus, the mitigation of metabolic acidosis by aspartate supplementation would positively impact the RSA. Despite attenuated blood pH reduction, aspartate supplementation did not affect the blood lactate concentration. Previous studies reported that aspartate supplementation reduced the blood lactate concentration during submaximal exercise [3,6], while a study reported no effect on blood lactate during exercise until exhaustion [7]. It is speculated that aspartate would reduce blood lactate during submaximal exercise, but not during exhaustive or maximal effort exercise. Further examinations including various intensities are needed. We speculated that $\overset{\cdot }{V}$O_2_ during the exercise would be higher in the ASP trial than in the PLA trial because aspartate facilitates the TCA cycle flux and increases fat oxidation [2]. Also, if aspartate mitigates exercise-induced blood pH reduction, the reliance on buffering action via HCO_3_^−^ would decrease, leading to attenuated $\overset{\cdot }{V}$CO_2_ output. However, we did not observe any difference between trials on cardiorespiratory data. We also speculated that plasma catecholamine concentrations may be altered when the contribution of carbohydrate/fat metabolisms is changed by the facilitation of the TCA flux, but there was no significant difference between trials.

Another potential factor of the positive impact is the involvement of metabolites of aspartate. Our results showed that the plasma levels of aspartate metabolic pathway members (glutamate, alanine, citrulline, and phenylalanine) [19] were also higher in the ASP than in the PLA trial. Glutamate and citrulline are transformed into arginine [20], increasing nitric oxide production and blood flow, and thereby possibly improving aerobic and anaerobic exercise performance [21]. However, the plasma arginine level did not differ between trials, meaning that the above mechanism related to arginine would not contribute to the improved RSA in the present study. The plasma BCAA (i.e., valine, leucine, isoleucine, and total BCAA) levels were also similar between trials. Thus, the improved RSA appeared to be independent of BCAA, which is a commercially available performance-enhancing supplement. In addition, aspartate, alanine, and glutamate are potentially transformed into oxaloacetate, which can be a gluconeogenetic substrate. However, no significant difference in blood glucose levels during RSA between the ASP and the PLA trials was observed.

Given that evidence, the improved peak cadence during the second exercise set in the ASP trial may be associated, at least in part, with the mitigation of blood pH reduction, probably attributable to the acceleration of the TCA cycle, as supported by a study demonstrating the facilitated TCA cycle flux and fatty acid oxidation by aspartate supplementation [2]. The plasma aspartate concentration showed a marked increase in the first exercise set and then a gradual reduction until the third exercise set, although we expected to maintain a high plasma level during entire the exercise by consuming the supplement twice. Considering that high-intensity exercise (>70% $\overset{\cdot }{V}$O_2_max) delays gastric emptying rate [22], the second supplementation would not contribute to increasing plasma aspartate concentration during the exercise. In addition, differences between trials were observed at different time points for plasma aspartate (the first and second exercise sets), blood pH (the third exercise set), and peak cadence (the second exercise set). The reasons for inconsistencies are unclear, but one possible explanation is that the time courses of blood and intramuscular aspartate concentrations may differ.

At 60 min after exercise completion, the ASP trial evidenced a significantly higher blood pH, HCO_3_^−^, and base excess than the PLA trial, suggesting that aspartate supplementation facilitates recovery from fatiguing exercise. Rapid pH recovery after a single session of exercise allows athletes to maintain high-performance levels during consecutive (multiple) games or training sessions because acidosis is considered to be one of the factors of fatigue during repeated-sprint exercise [10]. In contrast, the serum myoglobin concentration (an indicator of exercise-induced muscle damage) did not differ significantly between the trials. The cycling exercise used in the present study mainly consists of concentric muscle contraction, which is generally known to be less damaging compared to running exercises involving eccentric muscle contraction [23].

The present study had some limitations. Firstly, we did not control the physical activity level and diet pattern on the day before experimental trials, although participants were asked to avoid intense exercise, caffeine, alcohol, and supplements. In addition, we evaluated the acute effect of aspartate supplementation (2 × 4 g aspartate) or placebo supplementation on the trial days. Therefore, the impact of daily aspartate supplementation on exercise performance during consecutive days of training (e.g., multiple days of competition, training camp) remains unclear. Also, we utilized a relatively small dose of aspartate. Although there is evidence that smaller doses of aspartate show positive effects [5], most previous studies suggested that doses > 10 g of aspartate are effective on endurance performance [1]. Our data showed the positive effects of 8 g of aspartate (4 g twice per trial) on peak cadence in the repeated sprint. However, the dosage of aspartate was unified for all participants, and it was not manipulated individually based on body mass or muscle mass. Also, the participants ingested aspartate twice at different time points (before the first and second exercise set). Thus, further research is required to determine the optimal dose and appropriate timing of ingestion of aspartate. Furthermore, in the present study, the evaluation of RSA was limited. In particular, we were not able to determine the recovery of exercise performance after completing the third exercise set. This will be meaningful since the ASP trial presented a significantly higher blood pH 60 min after completing the exercise (vs. PLA trial). Unfortunately, we did not measure blood ammonia concentrations, which are considered as another potential factor for the performance-enhancing effect of aspartate supplementation [1]. Aspartate accelerates ammonia elimination in the urea cycle, and attenuates exercise-induced hyperammonemia, and may delay the fatigue. Further examination including the measurement of blood ammonia concentration is needed. Lastly, the participants in the present study were only male and were not athletes. As discussed above, aspartate may affect the RSA through the metabolism [1,2]. Since several studies reported sex differences in substrate metabolism [24], females also needed to be tested for the effects of aspartate supplementation on RSA. The participants were healthy active males but not athletes or well-trained because we could not recruit a sufficient number of homogeneous athletes with a similar training background.

## 5. Conclusions

Aspartate supplementation improved peak cadence during the second exercise set of repeated-sprint exercise compared with placebo supplementation. However, the performance-enhancing effect was limited, since no significant effect was observed for the mean power output throughout the exercise session. Aspartate is naturally found in general foods and no issues were reported in the other clinical studies. Thus, aspartate would be a safety supplement for sports athletes.

## Figures and Tables

**Table 1:** Cardiorespiratory responses during repeated-sprint exercise with aspartate (ASP) and placebo supplementations (PLA).

	Set 1	Set 2	Set 3
HR (bpm)			
ASP	155 ± 3	157 ± 3	158 ± 3
PLA	154 ± 4	156 ± 4	156 ± 4
$\overset{\cdot }{V}$O_2_ (mL/min)			
ASP	2128 ± 78	2137 ± 86	2128 ± 86
PLA	2120 ± 85	2078 ± 92	2089 ± 100
$\overset{\cdot }{V}$CO_2_ (mL/min)			
ASP	2238 ± 83	1991 ± 84	1914 ± 78
PLA	2245 ± 88	1912 ± 80	1875 ± 92
$\overset{\cdot }{V}$E (L/min)			
ASP	80.1 ± 3.8	83.1 ± 4.2	82.9 ± 4.8
PLA	83.2 ± 3.8	82.7 ± 4.3	82.5 ± 4.7

Mean ± SE. HR: heart rate. $\overset{\cdot }{V}$O_2_: oxygen consumption. $\overset{\cdot }{V}$CO_2_: carbon dioxide production. $\overset{\cdot }{V}$E: minute ventilation.

**Table 2:** Blood lactate, glucose, plasma catecholamine, and serum albumin concentrations immediately before and after each set of repeated-sprint exercise with aspartate (ASP) and placebo supplementations (PLA).

	Baseline	Pre-Set 1	Post-Set 1	Pre-Set 2	Post Set 2	Pre-Set 3	Post-Set 3
Lactate (mmol/L)							
ASP	1.2 ± 0.1	4.4 ± 0.4	12.3 ± 1.0	11.6 ± 1.0	14.1 ± 1.1	12.6 ± 1.1	13.0 ± 1.2
PLA	1.2 ± 0.1	4.4 ± 0.4	12.3 ± 1.0	11.9 ± 1.0	13.8 ± 1.0	12.7 ± 1.0	13.9 ± 1.0
Glucose (mg/dL)							
ASP	91 ± 3	87 ± 1	100 ± 4	99 ± 4	104 ± 5	105 ± 4	105 ± 5
PLA	90 ± 2	90 ± 3	98 ± 4	97 ± 5	105 ± 5	107 ± 4	108 ± 6
Adrenaline (pg/mL)							
ASP	76.9 ± 9.9	99.7 ± 14.0	251.4 ± 35.1	148.0 ± 24.4	346.6 ± 56.6	213.4 ± 38.2	400.6 ± 74.0
PLA	80.7 ± 10.9	113.3 ± 21.9	312.3 ± 58.4	189.3 ± 45.7	357.1 ± 62.3	218.7 ± 49.1	409.8 ± 71.1
Noradrenaline (pg/mL)							
ASP	334 ± 26	581 ± 41	2367 ± 174	966 ± 85	2770 ± 276	1091 ± 111	2738 ± 281
PLA	333 ± 37	528 ± 48	2432 ± 204	894 ± 96	2472 ± 262	1000 ± 97	2607 ± 253
Dopamine (pg/mL)							
ASP	10.6 ± 1.0	11.6 ± 1.2	41.5 ± 4.2	34.2 ± 3.9	79.6 ± 12.4	51.0 ± 7.2	93.9 ± 14.7
PLA	11.8 ± 1.3	12.0 ± 1.2	42.1 ± 5.4	31.8 ± 4.3	68.9 ± 10.8	44.6 ± 6.8	80.9 ± 12.2
Albumin (g/dL)							
ASP	4.65 ± 0.05	4.92 ± 0.04	5.26 ± 0.05	5.13 ± 0.06	5.30 ± 0.06	5.15 ± 0.07	5.28 ± 0.06
PLA	4.70 ± 0.04	4.96 ± 0.04	5.31 ± 0.06	5.20 ± 0.07	5.37 ± 0.07	5.24 ± 0.06	5.35 ± 0.07

Mean ± SE.

**Table 3:** Plasma amino acid concentrations immediately before each set of repeated-sprint exercise with aspartate (ASP) and placebo supplementations (PLA).

	Baseline	Pre-Set 1	Pre-Set 2	Pre-Set 3
Aspartate (μM)				
ASP	7.6 ± 0.6	45.3 ± 9.2 ***	24.2 ± 4.5 *	14.5 ± 2.9
PLA	7.7 ± 0.7	6.1 ± 0.8	6.5 ± 0.9	5.6 ± 0.7
Serine (μM)				
ASP	110.7 ± 3.1	103.3 ± 2.9	107.2 ± 3.4 +	103.2 ± 3.7
PLA	108.3 ± 3.7	99.8 ± 3.4	97.6 ± 3.0	95.4 ± 3.1
Glutamate (μM)				
ASP	98.9 ± 8.4	93.8 ± 7.8	97.9 ± 8.5 **	96.6 ± 8.0 *
PLA	104.0 ± 6.3	75.1 ± 6.2	67.8 ± 5.8	69.8 ± 7.7
Alanine (μM)				
ASP	344.5 ± 26.5	442.2 ± 26.2	644.1 ± 29.9 ***	641.7 ± 21.9 ***
PLA	329.9 ± 23.7	390.0 ± 21.6	494.4 ± 18.8	509.9 ± 16.7
Citrulline (μM)				
ASP	28.7 ± 1.4	24.4 ± 1.5	28.3 ± 1.7	28.2 ± 1.5 *
PLA	28.5 ± 1.1	25.9 ± 1.0	26.4 ± 1.1	25.0 ± 1.2
Phenylalanine (μM)				
ASP	59.1 ± 1.3	67.7 ± 1.3 *	64.5 ± 1.7 *	63.9 ± 1.6
PLA	60.5 ± 1.6	63.4 ± 1.7	61.1 ± 2.1	62.0 ± 2.1
3-Methylhistidine (μM)				
ASP	5.1 ± 0.2	5.1 ± 0.1	4.9 ± 0.2 +	5.0 ± 0.2
PLA	4.8 ± 0.2	4.8 ± 0.2	5.1 ± 0.2	5.1 ± 0.2
Arginine (μM)				
ASP	81.0 ± 3.4	83.5 ± 3.9	87.6 ± 4.4	86.1 ± 2.9
PLA	82.5 ± 4.8	85.8 ± 4.3	87.0 ± 4.4	87.2 ± 4.6
Valine (μM)				
ASP	237.4 ± 9.3	231.5 ± 8.0	217.5 ± 7.2	217.7 ± 7.4
PLA	232.9 ± 10.0	233.8 ± 10.0	216.5 ± 9.1	215.3 ± 9.2
Leucine (μM)				
ASP	130.7 ± 4.7	129.8 ± 4.2	122.5 ± 4.0	121.7 ± 4.0
PLA	128.9 ± 3.6	127.9 ± 3.1	120.2 ± 3.8	120.1 ± 3.4
Isoleucine (μM)				
ASP	68.3 ± 2.9	66.3 ± 2.3	63.3 ± 2.4	61.7 ± 2.4
PLA	65.5 ± 2.9	65.6 ± 2.2	62.0 ± 1.8	61.4 ± 2.0
Total BCAA (μM)				
ASP	436.4 ± 15.7	427.6 ± 13.6	403.2 ± 12.8	401.1 ± 13.2
PLA	427.3 ± 15.0	427.3 ± 14.2	398.7 ± 13.8	396.8 ± 13.4
Total amino acid (μM)				
ASP	2776 ± 64	2917 ± 53	3107 ± 59 **	3110 ± 60 **
PLA	2736 ± 53	2792 ± 50	2857 ± 36	2856 ± 44

Mean ± SE. BCAA: branched-chain amino acid. + *p* < 0.1, * *p* < 0.05, ** *p* < 0.01, *** *p* < 0.001 between trials.

## Data Availability

Data are contained within the article.
